# Oral lichen planus: A retrospective study of 110 Brazilian patients

**DOI:** 10.1186/1756-0500-3-157

**Published:** 2010-06-03

**Authors:** Mônica Ghislaine Oliveira Alves, Janete Dias Almeida, Ivan Balducci, Luiz Antonio Guimarães Cabral

**Affiliations:** 1 Department of Biosciences and Oral Diagnosis, São José dos Campos Dental School, São Paulo State University, São José dos Campos, São Paulo, Brazil; 2 Department of Social Science and Pediatric Dentisty, São José dos Campos Dental School, São Paulo State University (UNESP), São José dos Campos, São Paulo, Brazil

## Abstract

**Background:**

Oral lichen planus (OLP) is a chronic autoimmune disease characterized by multiple clinical presentations and a relatively high prevalence in the population. This retrospective patient record study investigated the profile of OLP in a group of Brazilian patients seen between 1989 and 2009.

**Findings:**

The clinical records were analyzed and data such as gender, age, race, clinical presentation of OLP, site affected, presence of symptoms and extraoral manifestations of the disease, smoking habit, and consumption of alcoholic beverages were obtained. Among the 1822 records of patients with oral mucosal lesions, OLP was identified in 6.03%. Of these, 76.36% were females, with a mean age of 54 years, and 85% were whites. The reticular form was the most frequent (81.81%). Extraoral lesions were observed in 32.72% of the patients and painful symptoms were reported by 50.90%. The cheek mucosa was the site most affected (92.72%) and multiple oral lesions were observed in 77.27% of the patients. Among patients with OLP, 18.18% reported a smoking habit and 29.09% the consumption of alcoholic beverages.

**Conclusions:**

This retrospective study showed a relatively high prevalence of OLP in the population studied, with a predominance of the disease among middle-aged white women and bilateral involvement of the cheek mucosa. Reticular lesions were the most frequent, followed by the erosive form which is mainly associated with painful symptoms. No relationship with tobacco or alcohol consumption was observed.

## Background

Lichen planus is a chronic autoimmune disease that involves a type IV hypersensitivity reaction to antigen variations observed in the mucosal lining and skin [1-3]. The estimated prevalence of the disease in the general population is 2% [1]. Fifty percent of patients with skin lesions also manifest oral mucosal lesions, and 25% of patients with oral lichen planus (OLP) present only oral lesions [3,4].

OLP shows a predominance among females and mainly affects adult patients between their fifth and sixth decade of life [4-7]. According to Xue *et al *(2005) [4], 25.8% of patients with the disease are smokers and 24% consume alcoholic beverages. The most frequently involved oral sites include the mucosa of the cheek, tongue and gingiva. The mucosa of the palate and floor of the mouth is rarely affected [4,8,9]. Extraoral lesions are mainly found on the skin and cutaneous appendages and especially develop in the flexor regions of the legs and arms and on the nails. Other mucosal sites include the genitalia, esophagus, larynx, scalp, and conjunctiva [2,10].

The clinical features of OLP in the oral mucosa are generally polymorphic and usually consist of bilateral and/or multiple symmetric lesions, with manifestation of associated clinical patterns [1-3,10]. Alternation between phases of exacerbation and quiescence has been reported [2]. OLP is classically divided into six forms according to Andreasen (1968) [11]: reticular, plaque-like, papular, atrophic, erosive, and bullous. The reticular form is the most common, followed by the erosive form. The latter manifests painful symptoms and has been associated with possible malignant transformation of lichen planus [4,6,7].

OLP is diagnosed clinically by means of a biopsy for histopathological analysis [2]. The classical microscopic features observed in the oral mucosa include hyperorthokeratosis or hyperparakeratosis, acanthosis, thickening of the spinous layer, liquefaction of the basal layer accompanied by the degeneration of keratocytes (hydropic degeneration), and lymphocyte infiltration of the lamina propria [1-3].

The present retrospective patient record study investigated the profile of OLP in a group of Brazilian patients with a diagnosis of the disease seen between 1989 and 2009.

## Methods

A cross-sectional, observational and retrospective study was conducted. The study was approved by the Ethics Committee of the São José dos Campos Dental School, São Paulo State University (UNESP) (protocol 019/2009-PH/CEP). Written consent for publication was obtained from the patients.

The sample consisted of patients with a diagnosis of OLP who had been followed up between 1989 and 2009. The clinical records of patients seen at the Stomatology outpatient clinic were reviewed for data collection. Records of patients with a diagnosis of lichenoid dysplasias or lichenoid lesions reactive to mechanical irritation and drugs, and incomplete or inaccurate records were excluded from the sample.

The following clinical data were obtained: gender, age, race, clinical presentation of OLP, site affected, and presence of symptoms and extraoral manifestations of the disease. Data regarding smoking habit and/or consumption of alcoholic beverages were also evaluated.

In the present study, the diagnosis of OLP was generally made based on the clinical aspects of lesions installed in the oral mucosae and sometimes confirmed by the analysis of lesions found on the skin, nails or other mucosa, if present. A biopsy was only obtained in atypical cases and the material was sent to the Laboratory of Oral Pathology for histopathological analysis.

## Results

Table 1 summarizes the profile of the patients studied. Among the 1822 oral mucosal lesions diagnosed during the study period, 110 (6.03%) were OLP lesions. Eighty-four (76.36%) cases were detected in females and 26 (23.63%) in males, with a proportion of 1:0.3.

**Table 1:** Profile of patients with OLP.

Profile	Women	Men	Total
	n = 84	n = 26	n = 110
	76.36%	23.64%	100%
Age (years)			
Mean	54.08	52.88	53.8
Range	26-97	22-74	22-97
Smoking	14	8	22
Alcohol consumption	20	12	32
Diabetes	5	1	6
Hypertension	18	4	22

**Figure 1:**
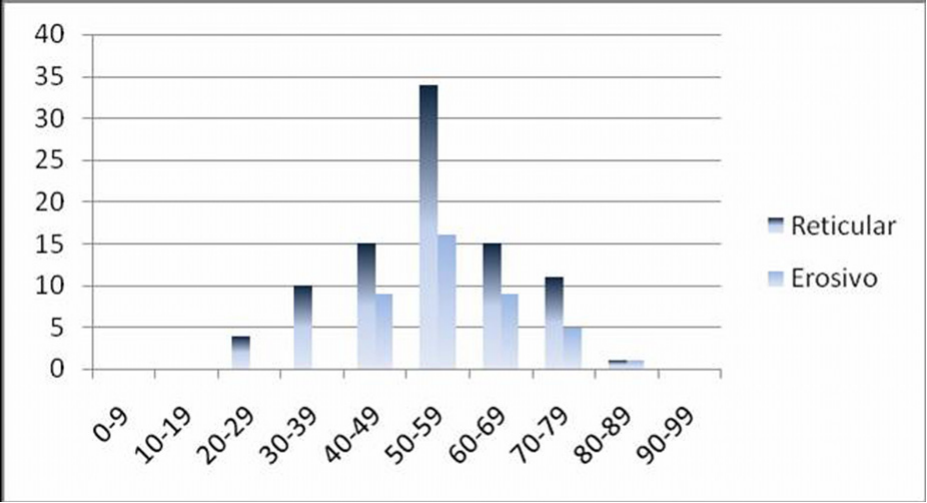
**Distribution of the 110 patients with OLP according to age**.

The mean age of the patients at the time of diagnosis was 54.08 ± 13.14 years for women and 52.88 ± 13.96 years for men [range: 22 to 97 years; Q1 = 45.75, Q3 = 63.00] (Figure 1). There was a predominance of the disease among whites (n = 94, 85%). Twenty-two (18.18%) patients with the disease were smokers and 32 (29.09%) consumed alcoholic beverages.

Most of the patients studied presented multiple oral lesions. The cheek mucosa was the site most affected (n = 102), followed by the tongue (n = 50), gingiva (n = 21), alveolar ridge mucosa (n = 9), and lip mucosa (n = 2) (Table 2).

**Table 2:** Frequency of involvement of oral sites in the two most frequent clinical forms of OLP.

Oral site	Reticular (n = 90)	Erosive (n = 46)
Cheek mucosa		
Bilateral	67	31
Unilateral	7	2
Tongue mucosa	30	19
Lip mucosa	1	1
Gingiva	14	7
Palate	1	1
Vestibular fornix	4	0
Alveolar ridge	6	2

Extraoral lesions were observed in 36 (32.72%) patients and were mainly detected on the nails (n = 12), wrist (n = 5), and ankle (n = 4).

The reticular form was observed in 90 (81.81%) patients and was associated or not with another form at the time of diagnosis. The erosive form alone was observed in 46 (41.81%) cases and was associated with the reticular form in 56 (Table 3).

**Table 3:** Differences between the two most frequent clinical forms of OLP

Variable	Reticular (n = 90)	Erosive (n = 46)
Gender		
Male	21 (23.33%)	7 (15.21%)
Female	69 (76.66%)	39 (84.78%)
Age		
≤50 years	29 (32,22%)	15 (32.60%)
>50 years	61 (67,77%)	31 (67.39%)
Symptoms		
Yes	45 (50%)	35 (76.08%)
No	45 (50%)	11 (23.91%)
Number of sites affected		
≤2	54 (60%)	28 (60.86%)
≥3	36 (40%)	18 (39.13%)

Pain was the most frequent symptom (47.27%). Among patients with painful symptoms, 76.08% had the erosive form associated or not with other forms. Isolated reticular lesions were found in the cheek mucosa and were asymptomatic.

## Discussion

The clinical characteristics of the patients studied here were similar to those reported in the literature, although some important differences were observed. A review of records comprising a period of 20 years showed that 6.03% of the oral mucosal lesions corresponded to OLP, a high prevalence when compared to the rate of 2% estimated for the general population by Anuradha et al. (2008) [1]. On the other hand, Pakfetrat et al. (2009) [12] reported a prevalence of 18.2% for Iranian patients, a rate three times higher than that observed in the present study.

A predominance of OLP among female patients was observed in the present study, in agreement with other reports [4,5,7,9,12,13]. According to Ingafou et al. (2005) [9], OLP mainly affects white patients. A predominance of OLP in the fifth, sixth and seventh decades of life was observed in the present study, in agreement with Ingafou et al. (2005) [9] and Hietanen et al. (1999) [7], although other studies did not show the expressive involvement of patients in their seventh decade of life [4,13].

There are no literature data indicating an elevated prevalence of smoking or alcohol consumption among patients with OLP compared to the general population [4,13], a finding also observed in the present study.

The cheek mucosa was the site most affected, followed by the tongue and gingiva, in agreement with other reports [4,8,9,12]. Extraoral manifestations were observed in 32.72% of the patients studied and exclusive oral lesion in 67.28%. According to the literature, 50% of all patients with lichen planus simultaneously present skin and oral lesions, whereas 25% present only oral lesions [14]. In contrast, other studies have reported cutaneous involvement in less than 17% of patients with OLP [12,14].

The reticular form was the most frequent, followed by the erosive form. These two forms were found to be associated or not with other forms, as also reported by other investigators [4,6-9].

Pain was the most frequent symptom and was observed in 47.27% of cases; of these, 76.08% had the erosive form of OLP. Similar results have been reported in previous studies [4,6].

## Conclusions

Observational retrospective studies have various limitations. However, the present investigation revealed a relatively high prevalence of OLP in the population studied, with a predominance among middle-aged white women and bilateral involvement of the cheek mucosa. Reticular lesions were the most frequent, followed by the erosive form which is mainly associated with painful symptoms. No relationship with tobacco or alcohol consumption was observed.

## Competing interests

The authors declare that they have no competing interests.

## Authors' contributions

MGOA analyzed, interpreted patient's data from the files and wrote the manuscript. JDA participated in the design of the research and helped to draft the manuscript. IB performed the statistical analysis. LAGC conceived and coordinated the research. All authors read and approved the final manuscript.
